# Tightening early childcare choices – gender and social class inequalities among Polish mothers in Germany and the UK

**DOI:** 10.1186/s40878-018-0102-6

**Published:** 2018-12-13

**Authors:** Karolina Barglowski, Paula Pustulka

**Affiliations:** 1 Technical University Dortmund, Institute of Sociology, Emil-Figge-Str. 50, 44227 Dortmund, Germany; 2 SWPS University of Social Sciences and Humanities, Youth Research Center, ul. Chodakowska 19/31, 03-815 Warszawa, Poland

**Keywords:** Gender, Parenting, Transnational migration, Childcare, Inequalities, Social protection, Polish migrants

## Abstract

Care for young children continues to highly influence the life chances of men and women, even more so when they are migrants. For migrant women, childcare remains a particular challenge when their kin are absent and the gendered norms of work and family life abroad diverge from what they have known in the country of origin. This article contributes to a deeper understanding of social class and childcare strategies of migrant women by combining two research projects with migrants from Poland to Germany and the UK. Accounts represented in this article depict the ways in which migrant mothers interpret and use the available childcare options, thereby highlighting how class-based resources are deployed and reproduced in two different welfare regimes. The comparative approach pursued in the article reveals that it is *neither* class *nor* national context that has a capacity to determine early childcare choices on its own. Instead, it is an intricate interplay of social protections’ availability, gender norms and social class, which together engender various childcare strategies.

## Introduction

From the inception of the gender lens into migration studies in the early 1980s (Morokvasic, 1984), the topic of childcare was seen as a burning issue for migrant mothers (Hondagneu-Sotelo & Avila, 1997). Since then, literature has extensively dealt with (transnational) care provisions pertaining to the challenges linked to childcare (Zontini, 2006; Ryan, 2007; Ryan, Sales, Tilki, & Siara, 2008; Kofman, 2012; Baldassar & Merla, 2014; Barglowski, Bilecen, & Amelina, 2015; Barglowski, Krzyżowski, & Świątek, 2015; Pustułka, 2016). These are undergirded by the fact that relocating abroad largely entails changes in women’s social relations and mothering practices, often devoid of support from the kinship members as a direct consequence of mobility. At the same time, studies have also shown that in spite of the changing gender norms, women still carry the main burden of securing childcare. This especially holds for the early motherhood period which engenders manifold social risks, because children’s early years are seen as requiring intensive maternal care. As a result, motherhood often hinders women’s labour market performance and deteriorates their economic position (Miller, 2007; Pustułka, 2015). The ways women strategize their childcare choices in the bounds of their social class is thus of utmost relevance for their future life chances. Although it has been shown before that mothers differently use childcare facilities according to their class (Lareau, 2000; Jensen, 2010), these insights have not been sufficiently applied to the distinct realities of migrant motherhood (see also, Fibbi & Truong, 2015). In this article, we therefore look at how social class patterns early childcare choices of women from Poland who have migrated to Germany and the United Kingdom. The comparative approach perused in this article henceforth looks at how social class plays out in two, different welfare regimes. In doing so, growing interest in various issues around migrant care, such as maternal capital brokering (Erel, 2010), access and construction of social networks post-migration (Zontini, 2006; Ryan, 2007; Ryan et al., 2008), transnationalisation of care practices (Kofman, 2012; Baldassar & Merla, 2014) and access to formal and informal care (Barglowski, Bilecen, & Amelina, 2015; Barglowski, Krzyżowski, & Świątek, 2015) are accounted for. Specifically, we pay attention to how migrant mothers’ capital shapes parenting of young children below the age of six. The 6-year-old mark demarcates the end of a particularly care-intensive phase in children’s lives and usually signifies a transition from home-based to school-led socialization and care. Importantly, this temporal frame tends to also coincide with a period when people make greatest advances in their professional lives, thus putting women at a disadvantage of facing doubled work and family challenges. Consequently, parenting young children is also tangibly interlinked with the scope and span of social protections available in a given welfare regime.

Our intention in this article is to combine insights from migration scholarship and inequality research, thereby deepening the understanding of the differential life chances of people in the context of migration. What we observe is that studies in the aforementioned two realms both emphasize the central role of gender and class in caregiving, yet these are often not brought into dialogue with each other. For instance, despite inequality research demonstrating the role of class in mothering and care (Lareau, 2000; Jensen, 2010), migrants are predominantly analysed through an “ethnic lens” which ignores the heterogeneity of class. This article thus aims to capture the diversity of migrant subjectivities and class-based differences, specifically by focusing on how social class of migrant mothers affects their early childcare provision options and choices.

## Social class and migrant mothers

Like in other areas of social life complex and often contingent processes of migrants’ capital evaluation and recognition in the immigration country severely impact on the broader situation of mothers representing different social classes. We formulate class analytically here from the perspective of Bourdieu’s forms of capital, and its modes of reproduction (habitus, fields, taste and distinction; Bourdieu, 1986). In doing so, we acknowledge that opportunity structures produce and are produced through individual’s actions that draw differently on capital, assets and resources (=CAR’s, see Savage, Warde, & Devine, 2005). The approach focused on CARs acknowledges the complexity of class in the process of migration, calling for analytical sensitivity to the spatial contexts that pattern the formation and validation of economic, social and cultural capital across various contexts. As Erel and Ryan (2018) argue, such a perspective acknowledges both migrants’ agency and the constraints of national and transnational social structures which result in complex trajectories of social mobility. Empirically, we conceive of working-class positions as those occupations which correspond to blue-collar jobs. Such employment usually does not require formal training and is generally less secure and worse paid. Blue-collar job holders usually possess localized forms of capital and tend to rely on resources from co-ethnics in the destination country or their relatives in the sending country. Middle-class migrants tend to hold white collar jobs, which require formal education. Networks with co-ethnics and their family members in their emigration country are not part of their CARs to the same extent as they are for working-class migrants (Lopez Rodriguez, 2017).

Although we have used here formal education and employment status as a straightforward marker of class position, the focus on CARs entails that peoples’ incorporated forms of capital or “habitus” (ways of thinking, saying and acting) are equally important parts of their class position (Bourdieu, 1986). These habitual dispositions are acquired and reproduced in social contexts and guide peoples’ orientation of practice (Bourdieu, 1990), which is imbued by intersectional hierarchies. The interplay between class and gender is the most important for this work. The amassed forms of capital, which cluster into certain class positions, are usually inherent to the nation-states, with concomitant forms of recognition and value. As a result, migration may mean that cultural capital is devalued (Weiss, 2005) and gender norms diverge (Amelina, 2013), often effectively limiting parents’ and children’s future life chances (Pratt, 2012). In this view, many CARs cannot be simply transferred from one country to the other (Erel & Ryan, 2018; Nowicka, 2014).

As we have argued elsewhere (Barglowski, 2018; Pustułka, 2016), social class distinctively situates Polish women’s maternal choices. In this context the strategic importance of networks has been also emphasized as bound to Bourdieu’s (1986) mechanism of “capital conversion”, according to which lacks in one form of capital can be offset by other forms of capital. Those able to strategically deploy social capital may have a better chance to obtain a desirable effect and compensate for shortcomings in knowledge and experience needed for succeeding within the immigration countries’ important social institutions. As shown by Lopez Rodriguez (2017) educational decisions made for migrants’ children in London were based on mothers’ initial class status in the home country despite the objective class losses experienced by the Polish women upon mobility. It can be expected that immigrant mothers of young children - who are frequently isolated and confined to local networks (Pustułka, 2016) - find it particularly hard to ascertain legitimized ways of taking advantage of their CARs. The interrelationship between certain types of networks (“ethnic”, local or transnational), their quality (weak/strong) and the resources acquired through them is though by no means straightforward. Rather, as Ryan (2007) has emphasized, migrant women construct and access a variety of networks which provide them with distinct resources. In particular, with regard to employed women, the author has shown that professional networks are often favoured over “ethnic” ones, which also carry risks around rivalry and distrust. It is thus necessary to develop a subjective perspective on migrant mothers’ diversified needs and strategies in the context of class-based opportunity structures and subjectivity.

In the upcoming analyses, we account for migrant women’s varied understandings of their own subjectivities as mothers, akin other roles of workers. Combating the ethnic lens, we acknowledge women’s class-dissimilar opportunities of accessing childcare options. The transnational dimension comes to the fore not so much in that women’s class positions change through migration, but rather in the varied capacity for having one’s capital valued and legitimized in different contexts. Due to the better standardization systems for higher education certificates, those with vocational and technical trainings can find themselves at a cross-cultural disadvantage, yet may also benefit from more work opportunities in secondary employment sector for low-skilled labour abroad. To some degree these uncertain career results concern also academic professions, since training, for instance for teachers and lawyers, is based on nation-specific knowledge. However, for professionals, migration effects are less tied to the formal cultural capital, which is universal, but rather correspond to their incorporated and habitual dispositions, such as ways of thinking, speaking and acting. The latter make them similar to non-migrant academic middle-classes (Barglowski, 2018). In effect, migrant mothering is a key example for the wider issues of how capital permeates the reproduction of class. By conjuring markedly different childcare choices and availabilities of social protection, CARs prompt specific outcomes for both present and future prospects of migrant mothers and children.

## Gender norms and welfare regimes

Mothering, which is understood as discourses and practices of emotional labour purported for the preservation, nurturance and training of children for adult life (Hondagneu-Sotelo & Avila, 1997), is intimately linked with care. It is central for the reproduction of inequalities in terms of class-related advantages and disadvantages (Lopez Rodriguez, 2017). Having childcare options means that a woman can decide on becoming a stay-at-home mother, using kin networks, hiring (ethnic/local) help, or taking advantage of institutionalized early childhood education and care (ECEC). Importantly, living in a transnational space often means that women have limited experiences of the welfare systems and social protection schemes in the receiving country, so they may resort to using a combination of multiple care resources. The choice of childcare provisioning strategy is highly consequential, since it not only determines the professional pathways and social mobility of migrant women but has also paramount influence on the futures (e.g. educational attainment) of migrant children (Turney & Kao, 2009). To reiterate, the focus on CARs acknowledges the interrelationship between structural opportunities and class-subjectivity. We believe that mothers’ desirable position cannot be deduced from her particular role (employment versus stay-at-home), but from the degree of freedom that mothers experience in taking their roles. As such, we do not see either type of motherhood/childcare choice as superior but rather argue that women have distinctive arrays of options in terms of their pre-existing status and CARs.

The analysed variety stems from the availability, affordability, desirability, and accessibility of childcare for migrant and non-migrant mothers with different class backgrounds, as well as the intersections between their labour market opportunities, forms of capital and childcare options. As the women’s care choices hinge on the “assemblage of social protection” (Bilecen & Barglowski, 2015), which operate in the given welfare regime, they also make it important to study the diversity of migrants’ needs regarding the provision and exchange of protection and thereby shifting the focus from narrow conceptualizations of ethnicity to addressing their entanglement and co-construction with gender and class. In general, mothers’ presence is often held to be of utmost importance for the development of children, especially for those below the age of three. This may endanger women’s professional careers and weaken their economic position. According to a recent representative survey research, as many as 78% of men and 75% of women in Poland agree that a woman who has young children should not work, while around 60% of the respondents claim that it is a woman who is ultimately responsible for the family (Krzaklewska et al., 2016). Thus, it is mostly mothers’ absence, through migration or circulation that is often perceived as threatening children’s development (Urbańska, 2015). With fathering less affected by social norms (Pribilsky, 2004), men are able to continue circular migration between countries without having their parenting skills questioned. Furthermore, qualitative data (e.g. Slany, 2008) reveal that Polish migrants are generally reluctant to delegate childcare.

Though, ideologically, non-delegating care might be preferred, there are also economic factors to consider. As stipulated by Ryan (2007), the absence of affordable state-run childcare – as it is the case in the United Kingdom – necessitates drawing upon shifting combinations of local and transnational networks and forms of social protection. In this sense, it is typical for female economic migrants who then transition to parenthood to call on their mothers for help and advice on their new-borns. This signifies thriving towards securing childcare that could allow both parents to return to the labour force, while simultaneously ensuring the teaching of the mother tongue, as well as passing on the tradition and values of the country of origin (Vasquez, 2010). Moreover, the diversification of care models corresponds with an interest in the physical presence of the trusted kin-carers (Mazzucato, 2008) and reinforces a traditional model of a multigenerationally co-residential family. This gender-centric pattern shapes migration debates, wherein women must always align with the requirement of co-residentiality with their children (Urbańska, 2015; Pustułka, 2012). In this context, Ryan (2016) has demonstrated that Polish women in the UK may become more sociable and forge weak local ties to have a childcare safety net on site. These are complimentary to the family-centric networks which are typical for Poles but may have become unavailable due to spatial distance. In other words, an intergenerational family contract of relying on kinship is unattainable, meaning that mothers have to develop explanations and strategies for both becoming a stay-at-home parent and delegating care.

According to previous studies, class intersects with gender and engenders certain configurations and normativity of labour market activities and maternal subjectivities (Lareau, 2000), but there is less attention to the class impact on mothering in migration settings. As will be shown in this paper, class-related childcare choices are embedded in different institutional contexts across the two welfare regimes, the Bismarckian one in Germany and the Anglo-Saxon^1^ one in the UK. As argued by Evers, Lewis, & Riedel (2005), both regimes explicitly focus on expanding the provision of pluralistic forms of childcare since the 1990s. However, the historical and cultural roots of the UK and German welfare regimes pertain to different images of families and gender roles.

Traditionally the Bismarckian welfare state likewise the Anglo-Saxon one was orientated toward economic development and a liberal economy. The key explicit difference appears to be that the German governance strongly supports a socially-desirable model of a family, wherein the division between women’s caring and men’s providing/breadwinning roles remains quite apparent. As such, welfare benefits tended to support either an exit or a non-entry of women onto the labour markets. In contrast, the Anglo-Saxon regime is typically a more ‘residual’ one, especially in a lesser focus on framing a particular family model and a forgone income transfer. The UK model is grounded on an economic liberalism and is often characterized as a ‘work-enforcing mechanism’ (Leibfried, 1996). Therefore, we can observe an increased investment in early-years education programmes, which are, in the UK, centrally-dependant on state funding from a top-to-bottom approach. In a bottom-up model, German cities and communities can incept a wider range of tailored solutions. While both systems have been strong in promoting economic development, the UK focus was less keen on supporting normative ideals of a singularly appropriate family life. Nowadays, we see that the governing body in the UK seeks to ‘manage the childcare market’ across family models, while Germany demonstrates commitment to subsidiarity and mixed approaches that foster the continuity of traditional care models. In particular, this has made a working mother and dual-career household model only recently acknowledged and somewhat promoted in the latter regime and effectively explains why the expansion of provisions in Germany proceeds at a slower pace (Evers et al., 2005, p. 204).

Within these different welfare regimes, migrant mothers strategize their childcare options in line with their social class. It should be noted that both Western welfare regimes differ from the Polish case. There, neoliberalisation ensued after the communist state and has been paired with rolling-back the state and resulted in the reinforcement of a traditional family model and decreased participation of women in the labour force (Pustułka, 2015). Following a brief review of the projects’ methodology, the findings are structured around the class positions of the Polish migrant mothers in the UK^2^ and Germany.

## Methodological approach and linking the two studies

While the presented analysis combines two separate research projects conducted by the authors, both the thematic similarities and the aligned qualitative approaches allow for a combination of results. The reasoning for expanding the dataset directly answers a call for more comparative work in migration research and yields itself to an additional in-depth analysis focused on a particular finding or aspect of the broader primary work undertaken (Heaton, 1998). For the purpose of this paper, the findings were brought together with the aim of reconstructing the impact of social class on the mothering practices pertinent to the early childcare provisions, which were observed in Germany and the UK as two major immigration countries of Poles (for a similar approach, see Moskal & Sime, 2016). Data collection in both studies relied on a combination of semi-structured and biographic interviewing methods. Coincidentally, the insights from the interviews were complemented by participant observations in respondents’ homes, as well as Polish schools and churches in both studies.

The interviews were meticulously transcribed and, in the first step, analysed with the use of open-coding. As for the second step, while Study 1 by Karolina Barglowski then proceeded to sequence analysis according to hermeneutic methods, which aim at the reconstruction of meanings that drive social action (Barglowski, Bilecen, & Amelina, 2015), Study 2 carried out by Paula Pustulka relied on the categorical case-by-case and cross-comparative analysis. The re-analysis of the data included in this article sought to explore the specific ‘orientations of practice’ (Bourdieu, 1986) that guide the ways in which mothers define their social roles regarding childcare and employment.

The Study 1^3^ was conducted between 2012 and 2015 as part of a joint research project (Faist, Bilecen, Barglowski, & Sienkiewicz, 2015). The findings in this paper draw on the subsample of 17 Polish migrants in Germany residing in two medium sized cities in North-Rhine Westphalia, among whom 4 were joint interviews with heterosexual couples. Respondents were between 33 and 67 years old. They had between one and three children, mostly in either kindergarten- or primary school-age. Five of them had a university degree, and 12 had vocational training, which however was not always recognized in Germany. Those who had their degree recognized (5), worked in more skilled jobs, such as gardeners and clerks, accounting here for the lower-middle-classes. Seven who had their degrees unrecognized for various reasons, were mostly employed in precarious and low skilled occupations, such as cleaners or construction workers and are considered here as working-classes. People’s arrival times varied from recent period to more than 20 years ago.

The Study 2^4^ focused solely on Polish migrant women with children. The conducted core 37 interviews constituted a basis for a doctoral thesis completed at Bangor University. The data collection honed in on rural and suburban areas of the United Kingdom (Wales and North-West England) and Germany (Hessen and North-Rhine Westphalia). The socio-demographic characteristics of the respondent group point to an age average of 37.9 years, though included women who ranged from 23 to 64-years of age. Out of the 37 interview-partners, 16 had a university degree a further 12 had either technical training or an A-level diploma, while the remaining nine had vocational qualifications. All but two women were in paid employment (six worked part-time), and the occupational status of the interviewees varied from low-level to quite prestigious positions. All but two participants remained in ethnically homogenous heterosexual marriages. On the whole the women were parents to 74 children. As many as 18 respondents had at least one child under the age of 5 at the time of the interview. Otherwise children’s ages varied from several months to adulthood. All participants arrived in their respective destination countries between 1980 and 2010, their average length of stay amounting to just under 9 years.

## Nation-bound resources and mothers’ restricted options in less-privileged households

Reflecting what researchers called a ‘survival strategy’ (Slany, 2008), some of the respondents found themselves escaping from marginalization, destitution and debt through migration. After coming to a foreign country and securing an income that helped the family ‘crawl out’ of the social margins, migrants display a discernible and explainable preponderance for subjectively improved view of their class position:

We couldn’t afford anything in Poland and were always in debt. My work, degree or not, never got us anywhere, I brought no money in. We just had to leave because there was no chance we’d get a house or anything. And here we came and I cleaned in offices and washed dishes and made sandwiches and we could live off that […] Hourly-paid work is easier to fit into your schedule with children. I did that, my husband worked nights and we got by. Then we got benefits, yes, things got much easier for me (Agata, 46, UK, 3 children aged 9 & 6, pregnant at the time of the interview)

Gender intersects with social class and compels specific ways in which the normativity of labour markets and maternal subjectivities collide. In that sense, destination regimes often capitalize on mothers as workers, yet tend to relegate women – unlike men - from working-classes into biological reproduction settings of traditional families by offering them fewer good opportunities (Pustułka, 2015). This concerns the migration to Germany to a particular degree because people expect that ‘in Germany one income is enough to live’ (Barglowski, Krzyżowski, & Świątek, 2015) and that institutions support the non-working housewife model through mechanisms of beneficial taxation and relatively long and generous maternal leaves. For many mothers of the working-classes, this institutional infrastructure offers what they consider ‘good care options’:

Most of the mothers I know stay with the kids for a long time. More so than I see women doing in Poland. State supports it and I think it’s great. It’s their early years, you can’t put a price tag on this and nobody will be as good for them as a mum. I know it sounds harsh, and I think that at 3 they should start kindergarten, but still, before that I’m home even if it’s bad for my career, I don’t care (Aga, 24, Germany, 2 children aged 4 & 1)

Some might say that I must be dissatisfied just sitting at home. True, it is not that easy; you just have to get into the spirit of doing it. […] You need to find your own fulfillment. I want to do it; it gives me pleasure. (Aneta, 43, Germany, 3 children aged 14, 10 & 8)

Compared to the life situation in Poland, the possibility to quit waged employment is held to be a privilege in Germany, as well as a signifier of a migration success. As also evidenced by White (2011) for Poles in UK, having more time for one’s family is a common motivation behind migration projects. Although gender norms are changing, latest when couples have children, many couples practice a traditional male earner and female caregiver model (Pfau-Effinger, 2004), particularly in the Polish migrant and more religious households (Mazurkiewicz, 2018). This orientation concerns working-class and lower-middle-class mothers in particular because their constraint labour market position renders their waged employment less rewarding in economic terms but also with regard to their “self-fulfilment” through employment.

The gendered norms in Poland, which the mothers take with them to the immigration country, explain why many women see staying at home as a good decision. While this is explicitly supported by the German welfare state orientation, the income discrepancies in the UK allow for this type of solution as well. Notably, the interviewed women largely grew up with the ethics of care in femininity models (Gilligan, 1982), which Titkow (2007) noted resulted in a gendered socialization of Polish children based on raising girls to be ‘caring’, and having care understood as a female obligation and task that gives women’s lives a meaning (pp. 240–241). The respondents had mothers who experienced double-burden of work and household duties, yet nonetheless occupied inferior social positions. It is thus not surprising that some women evaluate the decision to quit employment as a privilege and a ‘migration success’. Among others, Aneta expressed doubts about the potentiality of having three children had she and her husband stayed in Poland.

Gendered norms imported from the sending country are usually not at odds with the conditions women find after immigration to Germany and thus respondents seem to aspire to the ideal of a traditional family. Patrycja, based in a small-town in Germany, has put it as ‘feeling blessed to be able to stay home’. In contrast this means that women, in particular those in blue-collar occupations, who go back to work, feel inadequate and resented. Kasia reported: ‘my friends don’t approve and my mum doesn’t approve and I also felt bad but we couldn’t afford for me to take more than a year of maternity leave’. As former regimes made it normalized for Polish women to work, Kasia experiences negative ascriptions of work not so much because of her Polish background’s expectations but rather due to the explicit presumption that migration should have improved the household’s economy in a way that made her employment unnecessary.

The more liberal welfare state regime of the UK does not support this kind of housewife model to the same extent as Germany (Pfau-Effinger, 2004). The respondents in the UK therefore made a number of references to the welfare state, citing tax credits, child allowance, and maternal benefits as much needed support. Not many of them, conversely, directly talked about the gendered premises on the ideal family models, focusing rather on the universal, pragmatic solutions. A stark difference between both countries lied in the tremendous costs of childcare in the UK where a fee for a registered childminder or a nursery vastly exceeded the possible earnings of the blue-collar women:

With the [childcare] prices here, me working actually makes no sense because it would be like 50 pounds left of my salary after we paid for the nursery. With what we get from the state and what my husband makes, we can make do. Plus I can spend time with the little ones, take some time off. We once considered a Polish nanny to come part-time but it would still be too costly, so I’ve been staying home (Daria, 27, UK, 2 children aged 6 & 1).

While women usually subordinate their own labour market activities to the routines of their spouses and children, working-class and some of the lower-middle-class women often experience much more exploitative lifestyles, referring to very long working days. This was the case of Paulina, who immigrated to Germany in 2011 after her husband has signed a working contract with a temporary employment agency (*Leiharbeitsfirma*). Since then, he works as a construction worker and she holds cleaning jobs in the early morning and late evening hours, as in those times her husband can take care of their children. She understandably interprets her 16-h-workdays as very tiring. In Poland, she has worked as a cook, but she assumes that her qualification would not be recognized in Germany. In Paulina’s experience, the case of being prevented from working in one’s learned vocation affects women more than men:

At the moment I don’t work, I mean there are many people here who do not work in their profession. The Germans in Germany maybe yes, but if it comes to those Poles who immigrated here, that is very rare that people work in their own profession. OK, men might do so, working in construction, but women? (…) When it comes to me, well I would like to work as a cook, but at the moment I cannot, because of the children and the tasks around them (…) if the children grow up, then I would like to work again, but now it's not the time, my son is 7 and my daughter will be 5 in December (Paulina, 33, Germany, 2 children aged 4 & 7).

As demonstrated above, while some women voluntarily select the stay-at-home mother route, those working-class mothers whose husbands’ income is not considered enough to keep the household functioning, experience a worsening of their situation after migration. That is mostly the case when their forms of capital lose value through migration, thereby limiting their labour market opportunities. As such, many of the women feel that they sacrifice their own careers for a better future for their children. These respondents usually doubt that their own professional situation will ever be as good as it has been in Poland, pointing at gendered inequalities within migrant families.

This way, class intersects with migration background and thereby amasses to a double-marginalization which has conspicuous effects on childcare. Quite frequently, these are tied to the strategies of reducing the feelings of dissonance as one tries to reconcile being a ‘good mother’ with limited social and cultural capital. Many of the working-class respondents in our studies seemed to be aware that their current situation conflicted with the idea of providing ‘a better future’ through mobility:

This neighbourhood we live in is not great. It’s quite bad actually, used to be a ghetto, a Pakistani suburb. We knew, I mean we got it when we came to rent this place, but that’s what we could afford, can’t leave, it’s, on the other side, it’s too expensive. […] We thought it would only be for a couple of years but we’ve been here forever. Rents are up […]. I don’t really like them [children] hanging out with the crowd here, it’s not a great crowd if you know what I mean. But it’s also that we can’t get them into any extracurricular things, like afterschool stuff, nothing beyond of what is free, so they hang around here (Matylda, 39, UK, two children aged 8 &10).

Both the working-class mothers and the more privileged group, have a clear vision of a ‘better life’ for their offspring. However, especially in the UK, they are often faced with a high level of consumerism and availability of goods on the one hand, yet confined to the ethnic spaces of social activities, on the other. This was visible for mothers reflecting on the early years of their transition to motherhood:

There are so many fancy things for mothers here. You drown in accessories and cute items when you have a baby, it was totally different from when I had the older ones [in Poland] – everything is cheap, you can get so much stuff in charity stores and in Primark or Matalan – always bargains! […] Food and going out is expensive here though – I got invited to some classes and meetings by my midwife but could not afford it to go. I mean, it’s strange because some of this is free, but getting a bus and then you need to get a coffee at the place where they hold it, it gets too much very quickly (Martyna, 44, UK, 3 older children and one daughter aged 4)

Martyna has not been able to take advantage of the locally offered post-natal support and while she regards the fact that her young child ‘has everything’, she simultaneously deprives, though involuntarily, herself and her toddler of opportunities to network with the community. The narratives show that gender norms of maternal dedication to raising children prominently feature in women’s decisions and elicit a preference for staying home and providing the best possible care. However, class-driven factors may swing a mother’s practices according to the welfare regime – legitimizing or de-legitimizing her choice. All in all, women from the working-class group decided to dedicate themselves to childcare before their children reached the milestone of being 1-year-old, and a considerable majority remained home longer.^5^ Even when women had some desire to return to the workforce, this was only possible when they otherwise complied with the existing and class-based gender and care models. However, there was a caveat that these strategies would be altered had a financial stability of the family became threatened.

### Universal forms of capital and mothers’ choices in more privileged households

Multifarious factors alleviate the challenge of procuring care for young children and the caring strategies employed by mothers located in the upper- and middle-class strata by virtue of their professional status abroad. A greater recognition of qualifications and generally more resources can usually assuage pressures from the Polish cultural gender norms for migrants with white-collar jobs. The women in this group tend to question traditional gender norms as means to define their identities and usually aim to organize childcare in a way that does not hinder their professional roles. In particular for educated women, who have invested considerable time and effort into their education and careers, workplace success is often a constituent of a satisfying life. In contrast to less privileged women, these migrants encounter better labour market opportunities. As an example Magda, who works in Germany as a teacher has said:

I connote my employment with self-fulfilment and not with the thought that I gain money for what I am doing at the end of the month. For me it is simple, working to have a satisfying life. That’s why I see the money I earn more like an addition to what my husband is earning. (Magda, 35, Germany, 2 children 3 & 1)

In Magda’s account we see that the male breadwinner model, which is an ideal for many less privileged women, is also prevalent among educated women, who often see their money, as “an addition to what my husband is earning”. Instead of seeing their income as crucial for the households’ economy, white-collar employment is akin to “having a satisfying life”. As such, many of the mothers, some of them representing a class of global professionals, see themselves actively fighting conventional gender norms:

There is nothing wrong with being a working mother. I actually believe that just the opposite is true, especially if you have a daughter – for her to only see you slaving away around the house, wasting away your education – that is not good, even if you can afford it […] I am not advocating killing yourself with 12-hour shifts when your child is just a few weeks old, but staying home for more than a year is just not for me. […] Obligatory kindergartens are there for a reason and it is not only to benefit the children. The option of being successful at your job is there – just as long as you want it (Hanna, 36, UK, 2 children aged 4 & 2)

Starting with the most evident financial differences between the two groups, the more privileged women could first and foremost decide on, and then financially afford, the form of childcare that appealed to them. On that note, with sky-rocketing prices of childcare in the UK, some women preferred to stay at home a while longer, often alternating with their partners in taking parental leave, and working part-time after hiring a childminder:

I am really so happy to be home with my children – to be the one to take care of them every night, morning and evening. Even though we now have less money because I am not working [full-time] I feel this time with them is an investment in their sense of security, trust and being loved […] (Bogusia, 34, UK, 2 children aged 5 &1)

As is shown in this excerpt, not all middle-class women are engaged in full-time employment, but, they deploy a variety of strategies to adjust their career advancement with their care commitments. Bogusia, for instance, deploys a typical middle-class orientation that Lareau (2000) has termed as “concerted cultivation”, referring to the active parenting and monitoring of their children, which allows the transmission of middle-class advantages from one generation to the other. Another strategy was to combine leaves with a professional reorientation, as Magda, for instance, has worked before her pregnancy as a consultant, but was increasingly unhappy with that and planned to undertake a teacher training during her maternal leave.

For 1 year I have been doing the teachers’ training (*Referandariat*). I only studied one subject in Poland, but here I need to have a second subject. So additionally (to practical teachers training), I need to study (at a university). I don’t know how I can handle that (…) It is very difficult and I need a lot of help from outside, Kita (full time kindergarten), and of course a nanny (*Tagesmutter*) (Magda, 35, Germany, 2 children aged 3 & 1)

In the same vein, Andzelika has used her time off with her child as a ‘sabbatical’, strategizing it with her migration project and using the time to take up a psychotherapist training. In this respect, academically educated mothers display a preference for institutional care to simultaneously pursue their professional careers. Magda and Andzelika made use of nannies and full-time kindergartens (*Kita*), while Sylwia made significant effort into finding a best ECEC institution in the area:

The *Kita* has a variety of experts on site – well-qualified teachers, a speech therapist, a dance instructor, and a sports’ coach… I am not sure even the best grandma could provide such a great environment, not to mention social development that happens in a group of children. My youngest will go to a nursery as soon as she’s one […] (Sylwia, 35, Germany, 2 children aged 5 years & 6 months)

The ‘world is your oyster’ attitude that academic middle-class mothers seem to promote in their childcare choices of special activities can be termed ‘cosmopolitan’, ‘expressed by an emotional and ethical commitment towards universalism, selflessness, worldliness and communitarianism’ (Skrbis & Woodward, 2007, p. 730). This renders maternal and family resources not nation-state-specific, but rather locates them in the realm of transnational forms of capital, which can be recognized in global and universal contexts:

You have to move forward for yourself and your children. Thinking that Poland was better is an illusion that is holding you back, you have to feel comfortable where you are, this will make you and your children successful (Justyna, 29, Germany, 1 child aged 6)

Additionally, the academic middle-class mothers usually do not spend time in the private sphere during early motherhood, but instead gather with other mothers from the same class-based or ethnic background, normally found in (international) playgroups and local meetings (for ‘expat’ mothers). Unlike the socio-economically constrained working-class mothers, they can select activities in line with their class status, going to, for instance, baby yoga and sing-alongs, as well as toddler read-aloud classes at the libraries. The mothers who had white-collar careers exude trust towards expert-certified maternal capital and investments, as they buy expensive equipment (strollers, bouncy chairs) and everyday items (organic food).

## Discussion and conclusion

In this article, we have explored the ways mothers strategize their own needs and those of their children and other household members as key dimensions of their class positions. The comparison of childcare orientations and practices of women from Poland in Germany and the UK have revealed complex intersections between availability of childcare options, parental class-based resources and the constitution of particular welfare regimes. In doing so, this article contributes to the recent research on the stratifying forces accompanying family migration (Kofman, 2018; Faist et al., 2015), thereby shedding light on the heterogeneity of migrant populations with regard to class (see also, Fibbi & Truong, 2015).

It has been demonstrated that care is a key dimension of migrants’ class, which is deeply interrelated with experiences of migration. This is shown in that many middle-class women see their migration as “an adventure” (Slany, 2008), which makes their migration appear to be less consequential with regard to deskilling or a loss of status. In our study, middle-class migrant mothers tended to benefit from robust forms of capital, assets and resources (CARs) that they could apply to the distinct realities of the immigration contexts. This corresponds with the knowledge about the CARs, which are easier to transfer between countries for the middle-class people, as compared to those with nation-bound resources (Weiss, 2005). Thereby, we have observed a convergence of social classes across both regimes in transnational spaces. This reproduction of class fundamentally shapes child care strategies, as women with white-collar job positions and background tend to find fitting combinations of early childcare options through networks, financial resources, knowledge and capacities, regardless of whether they found themselves in the UK or in Germany. In that sense, educated middle-class mothers fared better when it came to having options for reconciling family life with work. Conversely, working-class women see their migration as prompted by economic necessity or family reunification, which often contradicts their commonly high aspiration for upward social mobility (e.g. Lopez Rodriguez, 2017). In our studies, migrant women who held blue-collar jobs were less likely to be able to draw on their CARs and, ultimately, had to strategize childcare in the context of limited options. This often meant that they had to forgo their professional life and stayed at home with the children out of family’s necessity rather than as personal choice, largely because their own employment seemed to them to be less rewarding in economic and normative (“self-fulfilment”) terms (see also Mazurkiewicz, 2018). The constantly high normativity of traditional gendered division of care tasks is though also mirrored in the accounts of more privileged mothers. This is demonstrated by the fact that the vast majority of migrant mothers subordinate their own labour market activities to the routines of other members of their households (in general female employment is persistently seen as subordinated to males’, for Germany, see Auspurg, Hinz, & Sauer, 2017). Accordingly, the mothers in our studies mostly work when their spouses can take care of their children, thus their economic activity largely depends on their partners’ willingness to share caring duties and, independently of how high their income was, it had been usually regarded as “additional”. The workplace options of mothers are therefore limited because they need to align their schedules with children being away in the nurseries, kindergartens, schools, or at extracurricular activities.

The biggest class-based difference is that academic middle-class women often manage to get their children and themselves into the local networks and, thus, learn about the local system, either through the entrusted ECEC that predisposes their children to succeed educationally, or by networking with mothers who can advise them on what the best schools in the area are. While the specific configuration of formal and informal care depend on the opportunity structure provided by the welfare regime, the orientations and practices that mothers deploy are inherently shaped by their class background. As such privileged women flexibly take advantage of maternity leave, the length being dependent on the regulation of the distinct welfare regime, and a variety of local activities, such as baby groups, through which they build relevant networks and access social capital in accordance with the conditions they find after immigration. In our studies, they followed through with signing older children up for extracurricular activities like art classes, ballet, music lessons, sailing club or archery in both Germany and the UK. In both countries, they put strong emphasis on their professional role, while less privileged mothers have faced restricted labour market options. Accordingly, their cultural capital, both formal and incorporated (“habitus”), allow privileged migrant women, likewise middle class “native” women, to deploy their orientations towards good care, inter alia supporting bilingualism by balancing different kinds of childcare, both formal and informal, such as nurseries, kindergartens, childminders, friends and (paid) co-ethnic caregivers (so-called ‘ethnic nannies’), in order to either part- or full-time return to employment. Quite clearly, this time-space-bound and habitus-dependent strategy indicates that the institutions of early care education and care (ECEC) determine the potential of female employment. While the early childcare choices become tightened for all women, the CAR-related challenges of working-class mothers signify more struggles for this group. In that way, the known challenges with the transfer of capital upon international mobility (Erel, 2010; Erel & Ryan, 2018; Nowicka, 2014), are often exacerbated by motherhood.

It follows therefore that migrant caregiving is a function of gender norms in both the emigration and the immigration country, inadvertently tied to the class-based resources and subjectivity, with the welfare regime coming to play as an additional factor. By being inherently linked to the class-related opportunities, which are decisive for how migrant women juggle formal and informal social protection after migration, CARs and regimes need to be analysed together. At the end, class and gender norms, with a lesser role of the context, together shape women’s practices of employment and childcare.

## Footnotes

1 We use the classification of welfare states to outline key elements of the context in which mothers deploy their childcare strategies. We are we aware that classifications are analytical constructions based on overemphasizing similarity within a type and underemphasizing differences between types.

2 While we refer to the UK as the national counterpart context for Germany, it should be noted that the research for this work has been done in England in Wales. As such, it may omit contextual differences found in Scotland and Northern Ireland.

3 Karolina Barglowski has received funding for research from the German research foundation under the framework of 628 the Collaborative Research Centre 882, Bielefeld University.

4 Paula Pustulka has received funding from the 125th Anniversary Research Scholarship (2010–2013) awarded by Bangor University. The fieldwork in Germany was partially supported by a DAAD short-term visiting fellow grant (2011).

5 Note that many women organized childcare through ‘flying grandmothers’, i.e. by having their mothers as a live-in help during their child’s early years. While this phenomenon is a poignant sign of childcare transnationality, it has been left out of scope for this paper.
